# Metabolic Responses to Exercise and Nutritional Strategies in Type 1 Diabetes Using Automated Insulin Delivery Systems: A Narrative Review

**DOI:** 10.3390/metabo16070437

**Published:** 2026-06-23

**Authors:** Desirée Victoria-Montesinos, Inmaculada Llopis-Alonso, Ana María García-Muñoz, María Teresa Mercader-Ros

**Affiliations:** Faculty of Pharmacy and Nutrition, UCAM Universidad Católica de Murcia, 30107 Murcia, Spain; dvictoria@ucam.edu (D.V.-M.); icllopis@ucam.edu (I.L.-A.); mtmercader@ucam.edu (M.T.M.-R.)

**Keywords:** automated insulin delivery, artificial pancreas, type 1 diabetes, physical activity, exercise metabolism, carbohydrate counting, nutrition, metabolic biomarkers, metabolomics, hybrid closed loop, continuous glucose monitoring

## Abstract

**Highlights:**

**What are the main findings?**

- Automated insulin delivery systems improve glycemic control in type 1 diabetes, but exercise and nutrition remain metabolically complex scenarios that still require manual carbohydrate counting, meal announcement, exercise-target activation, and individualized decision-making.
- Exercise-related glucose responses depend on subcutaneous insulin kinetics, insulin on board, exercise modality, meal timing, substrate metabolism, and counter-regulatory hormonal responses, limiting the capacity of current single-hormone hybrid closed-loop systems to operate fully autonomously.

**What are the implications of the main findings?**

- Nutritional strategies in AID users should be personalized according to continuous glucose monitor trends, insulin on board, macronutrient composition, timing of the last meal, exercise modality, and platform-specific algorithm features.
- Future AID systems may benefit from integrating metabolic biomarkers, activity sensors, metabolomics, and adaptive algorithms but dual-hormone systems, digital twins, and metabolomics-informed personalization still require further validation in free-living conditions.

**Abstract:**

Background/Objectives: Automated insulin delivery (AID) systems have improved the management of type 1 diabetes (T1D), but exercise and nutrition remain challenging because they rapidly alter glucose flux, substrate oxidation, hepatic glucose output, insulin requirements, and fuel availability. This narrative review aimed to synthesize current evidence on the interaction between AID systems, physical activity, and nutritional strategies from a metabolism-oriented perspective. Methods: A narrative bibliographic approach was used to integrate evidence from clinical trials, observational studies, technical studies, consensus statements, and reviews involving people with T1D across different life stages, including pediatric, adolescent, adult, and pregnancy-related contexts, when available. The review focused on AID systems, exercise physiology, nutritional strategies, meal announcement, bolus adjustment, dual-hormone systems, metabolic biomarkers, and emerging metabolomic approaches. Results: AID systems generally improve time in range and reduce hypoglycemia across several user groups, although most exercise- and nutrition-specific evidence comes from adult and pediatric/adolescent cohorts rather than pregnancy-specific exercise studies. Exercise-related glucose responses remain highly dependent on user input, exercise modality, insulin on board, meal timing, and metabolic state. Planned exercise announcement, prandial bolus reduction before postprandial activity, and individualized carbohydrate intake remain key strategies. Biomarkers such as lactate, ketone bodies, non-esterified fatty acids, and counter-regulatory hormones may help explain interindividual variability and support future personalization. Conclusions: Nutrition and exercise management in AID users should be interpreted as a dynamic metabolic interface among exogenous insulin, endogenous counter-regulation, substrate availability, and algorithmic control. Emerging approaches, including activity sensors, adaptive algorithms, dual-hormone systems, digital twins, and metabolomics-informed personalization, may improve safety and reduce user burden, but several remain exploratory and require further validation in diverse free-living conditions.

## 1. Introduction

Type 1 diabetes (T1D) is a chronic autoimmune disease characterized by progressive pancreatic beta-cell destruction and lifelong dependence on exogenous insulin therapy [1]. In parallel with advances in insulin pharmacology and glucose monitoring, therapeutic management has progressively shifted from intermittent capillary glucose assessment and open-loop insulin administration to sensor-augmented pump therapy, including predictive low-glucose suspend systems, and subsequently toward integrated technologies that combine continuous glucose monitoring (CGM), subcutaneous insulin infusion, and algorithm-driven insulin adjustment [2]. Within this technological evolution, automated insulin delivery (AID) systems, also described as artificial pancreas or closed-loop systems, aim to improve glycemic control while reducing the day-to-day cognitive burden associated with diabetes self-management [3,4]. A central physiological constraint is that subcutaneous insulin absorption and action are substantially slower than endogenous pancreatic insulin secretion and suppression, creating a temporal mismatch when glucose turnover changes rapidly during exercise or after meals.

However, improvements in average glycemic control have not eliminated the instability produced by physical activity. Exercise may lower, stabilize, or increase glucose depending on intensity, duration, timing, insulin on board, nutritional state, substrate availability, and individual training status. Moderate aerobic activity commonly increases skeletal muscle glucose uptake and can precipitate hypoglycemia during exercise or later in recovery, whereas resistance exercise and high-intensity interval exercise may provoke catecholamine-mediated hyperglycemia, lactate accumulation, and subsequent glycemic variability [5]. For this reason, nutritional strategies, especially the timing and quantity of carbohydrate intake, remain central to exercise management, although their application has changed in the context of AID technology [6]. In real-world conditions, these determinants rarely occur in isolation. Exercise may coincide with recent prandial insulin delivery, high-fat or high-protein meals, low carbohydrate availability, alcohol intake, menstrual-cycle-related changes in insulin sensitivity, stress, illness, or recovery from previous training sessions, creating overlapping metabolic scenarios that current algorithms may not fully anticipate [7,8].

This limitation is particularly relevant because most currently available AID systems are hybrid rather than fully autonomous. They can modulate basal insulin delivery, suspend insulin, raise glycemic targets, or administer automated correction boluses, but users generally still need to announce meals and often need to anticipate exercise. Consequently, clinical outcomes depend on an interface between user behavior, nutritional decision-making, exercise physiology, and algorithmic control. Recent position guidance emphasizes that AID systems can improve safety around physical activity, but they do not remove the need for proactive planning, particularly when exercise is prolonged, postprandial, spontaneous, or performed with substantial insulin on board [7].

In this review, unless otherwise specified, the term AID primarily refers to currently available single-hormone hybrid closed-loop systems, which automate insulin modulation but still require user input for meal announcement, carbohydrate counting, and, in many cases, planned exercise. Dual-hormone systems, fully closed-loop approaches, glucose-responsive insulin technologies, digital twins, and artificial intelligence-based decision-support tools are discussed separately as emerging or experimental strategies.

Accordingly, the objective of this narrative review is to synthesize the evidence on nutritional strategies and physical exercise in people with T1D using AID systems, while reframing the topic through the metabolic pathways that determine glycemic responses [5,6,7]. The review focuses on clinical performance and safety, exercise-related glucose dynamics, carbohydrate and meal strategies, metabolic biomarkers, substrate metabolism, technological adaptations, special populations, psychosocial determinants, and implementation barriers.

## 2. Materials and Methods

A narrative bibliographic approach was adopted because the topic draws on heterogeneous evidence from clinical diabetology, nutrition, exercise physiology, metabolic biochemistry, behavioral science, and control engineering. Literature was identified from biomedical and multidisciplinary databases, including PubMed, Scopus, Web of Science, and Google Scholar, covering publications from January 2015 to May 2026. The final search was conducted in May 2026. Searches used combinations of terms related to T1D, automated insulin delivery, closed-loop systems, artificial pancreas, physical activity, exercise, carbohydrate intake, meal announcement, nutrition, dual-hormone systems, machine learning, metabolomics, metabolic biomarkers, lactate, ketone bodies, non-esterified fatty acids, acylcarnitines, substrate oxidation, and implementation. Search terms were adapted to each database, and articles written in English or Spanish were considered. Additional sources were identified from the reference lists of reviews, guidelines, position statements, clinical trials, technical papers, and metabolomic studies, and the approach was informed by the full body of literature synthesized in this review.

Consistent with this scope, studies were considered relevant when they addressed at least one of the following domains: clinical efficacy or safety of AID systems; glucose and metabolic responses to exercise under closed-loop or sensor-integrated insulin therapy; nutritional strategies around exercise; carbohydrate counting or macronutrient effects; meal announcement or automated meal detection; exercise-detection algorithms; dual-hormone or advanced control systems; metabolic biomarkers and metabolomic signatures; psychosocial outcomes; special populations; or real-world implementation. Studies focused exclusively on type 2 diabetes, gestational diabetes, open-loop insulin pump therapy without automated insulin adjustment, multiple daily injections without AID, animal models, or purely in silico approaches were not prioritized for the core clinical synthesis, although modeling and technical studies were considered when they informed emerging technological directions. The final synthesis prioritized evidence directly related to the interaction between AID, nutrition, exercise, and metabolism, while using broader diabetes technology literature only where necessary to contextualize clinical or implementation issues.

Because these areas are methodologically diverse, evidence was organized thematically rather than quantitatively. Particular weight was given to randomized and crossover trials, free-living studies, consensus statements, and studies that evaluated exercise or nutritional challenges under AID conditions, while reviews, modeling studies, and engineering papers were used to interpret mechanisms and emerging solutions. Quantitative information, including time in range (TIR), time below range (TBR), time above range (TAR), carbohydrate doses, sample sizes, and post-exercise glycemic outcomes, was extracted and reported when available. Reference management, duplicate checking, and screening were performed using Zotero software (version 7.0; Corporation for Digital Scholarship, Vienna, VA, USA) by two investigators (A.M.G.-M. and D.V.-M.), with uncertainties resolved by discussion.

## 3. AID Systems and Exercise-Related Glycemic Dynamics

Most evidence summarized in this section refers to single-hormone HCL systems, including commercial platforms that adjust insulin delivery based on CGM values, insulin delivery history, and user-entered information. Therefore, their exercise-related limitations should be interpreted in the context of subcutaneous insulin kinetics, residual insulin on board, manual carbohydrate counting, meal announcement, and the proactive activation of exercise or temporary targets when available.

Building on this technological background, hybrid closed-loop (HCL) systems have consistently demonstrated benefits over conventional pump therapy or sensor-augmented pump approaches. In adults with suboptimally controlled T1D, closed-loop insulin delivery improved time in range (TIR) and reduced exposure to hyperglycemia and hypoglycemia [9]. Nevertheless, comparative trials indicate that algorithm design matters. In the FLAIR trial, two HCL systems produced different glycemic profiles, showing that AID performance depends not only on the presence of automation but also on target settings, correction behavior, and the handling of meal-related insulin delivery [10]. This platform-specific behavior is clinically relevant during exercise. Commercial single-hormone HCL systems differ in their degree of automation, glucose targets, correction aggressiveness, and exercise-related features [11]. For example, MiniMed 780G allows temporary target activation and includes automated correction boluses; Tandem Control-IQ includes an Exercise Activity setting with a higher treatment range; Omnipod 5 includes an Activity feature that raises the glucose target and reduces automated insulin delivery; CamAPS FX provides user-adjustable modes such as Ease-off to reduce insulin delivery when lower insulin needs are anticipated; and Diabeloop DBLG1 incorporates physical activity declaration and preventive carbohydrate recommendations [12,13,14].

In contrast, studies of overnight closed-loop control illustrate that automation performs particularly well when glucose perturbations are relatively predictable and user input is minimal [15].

However, efficacy must be interpreted alongside safety. Insulin pump therapy and AID-related technologies can reduce severe hypoglycemia in selected populations, but any system based on subcutaneous insulin infusion remains vulnerable to diabetic ketoacidosis (DKA) when insulin delivery is interrupted [16]. In this context, predictive low-glucose suspend systems provide an additional protective layer by reducing nocturnal and exercise-related hypoglycemia risk [17]. Open-source or do-it-yourself AID systems have also reported favorable glycemic outcomes, although their regulatory status, training pathways, and professional support differ from those of commercial systems [18].

The challenge becomes more evident during exercise, because glucose trajectories can change faster than subcutaneous insulin kinetics. Dual-hormone approaches attempt to address this limitation by delivering glucagon in addition to insulin. In a randomized crossover trial, a dual-hormone artificial pancreas provided greater protection against exercise-related hypoglycemia than a single-hormone system during continuous and interval exercise [19]. Similarly, earlier bihormonal studies showed that autonomous adaptation can support glucose control during meals and exercise, while overnight dual-hormone control can reduce hypoglycemia exposure [20,21]. Nevertheless, dual-hormone systems introduce additional challenges related to device complexity, cost, glucagon dosis, avoidance of rebound hyperglycemia, and long-term usability. The development of stable liquid glucagon analogues, including dasiglucagon, has improved the feasibility of bihormonal approaches, and dasiglucagon has been evaluated in home-use bihormonal iLet studies [22,23]. However, chronic automated glucagon delivery remains less mature than currently marketed single-hormone HCL systems. Therefore, findings from dual-hormone studies should be interpreted separately from those obtained with commercial single-hormone HCL systems, as their physiological rationale, device requirements, and regulatory maturity differ substantially.

This continuing need for anticipation is most evident when exercise is announced versus unannounced. In young people with T1D, closed-loop control during and after unannounced physical activity reduced some hypoglycemic exposure but did not eliminate risk [24]. Moreover, postprandial exercise is especially challenging because prandial insulin may still be active when muscle glucose uptake increases. In adults with T1D, announcing postprandial exercise and reducing the meal bolus was more effective for limiting hypoglycemia than unannounced strategies [25]. These findings indicate that user-system communication remains necessary even when insulin delivery is automated.

Accordingly, sensor-based exercise detection has been proposed to improve AID responsiveness. Heart rate and accelerometer data can provide earlier signals of physical activity than CGM alone, potentially allowing algorithms to reduce insulin delivery before glucose decline becomes evident [26,27]. However, current systems must distinguish structured exercise from daily movement, emotional stress, illness, and sensor noise, and they must interpret the likely glycemic direction of activity for a specific user. This remains difficult because similar activity signals may have different metabolic consequences depending on recent insulin exposure, meal timing, and exercise modality [5,7,26,27].

Beyond the exercise bout itself, post-exercise management is also important. Adolescents with T1D may require additional glucose after moderate afternoon exercise in a biphasic pattern, with both immediate and delayed needs [28]. In addition, low-dose glucagon has been proposed as an alternative or adjunct to carbohydrate intake for preventing or treating exercise-related hypoglycemia, and the glucose response to low-dose glucagon appears to be preserved after exercise in insulin-pump-treated individuals [29]. These observations explain why delayed nocturnal hypoglycemia may occur even when glucose appears stable during or immediately after exercise.

Taken together, these studies indicate that the temporal relationship between exercise and meals determines the type of risk that AID can realistically manage. If activity begins soon after a meal bolus, the system can reduce future insulin delivery but cannot remove insulin that has already been absorbed. This distinction reflects the pharmacokinetic limitation of all subcutaneous insulin-based AID systems: reducing or suspending future insulin delivery cannot immediately reverse the glucose-lowering effect of previously delivered bolus insulin. In this context, meal bolus reduction and carbohydrate planning may be more important than automated basal modulation alone. Conversely, when exercise occurs several hours after a meal, the system has more opportunity to mitigate gradual glucose decline through target elevation, insulin attenuation, or suspension, although carbohydrate may still be required during prolonged aerobic activity [6,7,25,28].

Therefore, a practical distinction can be made between algorithm-manageable and user-manageable risk. Gradual glucose declines during planned aerobic activity may be partially addressed by current AID features, whereas rapid changes caused by recent bolus insulin, unannounced exercise, high-intensity intervals, or mixed meals often remain user-dependent. Clinical education should therefore avoid presenting AID as a substitute for exercise planning and should instead emphasize strategic use of automation, review of insulin on board, and interpretation of CGM trends in context [5,6,7,25,28]. Quantitative findings from exercise-focused studies illustrate the magnitude and variability of these effects. During prolonged aerobic hiking in AID users, activation of a higher glucose target without additional carbohydrate intake achieved higher exercise TIR than scheduled carbohydrate snacks (97.7% vs. 83.7%) and markedly lower post-exercise TAR (0.9% vs. 13.2–19.2%) [30]. In adults using MiniMed 780G during sustained cycling, fractionated isomaltulose intake during exercise achieved a TIR of 96.3% compared with 76.1% after a single pre-exercise dose, with minimal TBR in both conditions [31]. In the Omnipod 5 randomized trial, exercise TIR ranged from 87.8% to 92.3% depending on Activity Feature timing, although post-exercise TAR increased when the feature was activated earlier [13]. By contrast, in the free-living RAPPID study, mean TIR during unstructured physical activity was 69%, hypoglycemia occurred in 20% of sessions, and no significant differences were observed between MiniMed 780G, Control-IQ, and CamAPS FX, highlighting the gap between controlled protocols and real-world use [12].

To summarize the main exercise-related challenges that remain clinically relevant for users of automated insulin delivery systems, Table 1 presents the dominant physiological mechanisms, their practical implications, and the representative evidence supporting each scenario.

## 4. Metabolic Determinants of Exercise Responses During Automated Insulin Delivery

### 4.1. Pathway-Oriented Interpretation of Glucose Flux

**Figure 1:**
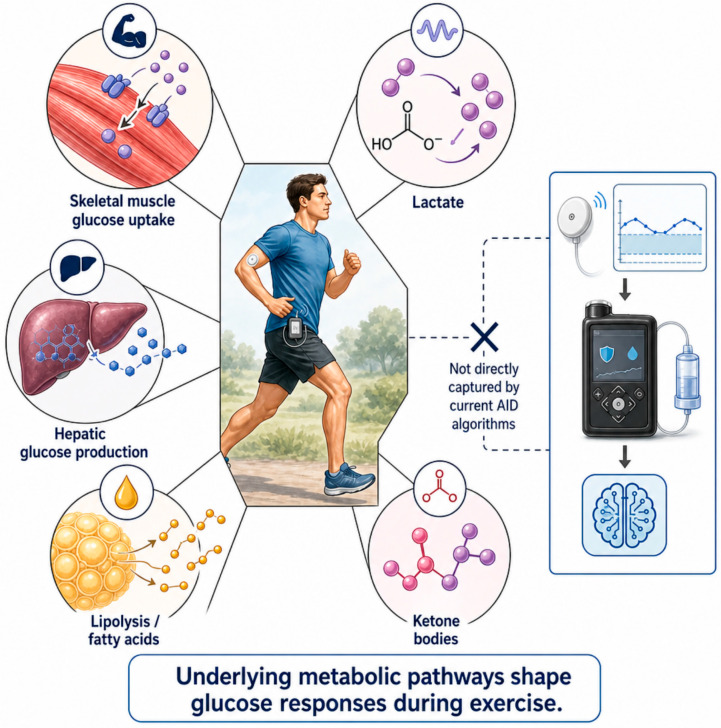
Metabolic determinants of exercise-related glucose responses during automated insulin delivery in type 1 diabetes. The figure summarizes the main physiological pathways that influence glucose responses during and after exercise, including skeletal muscle glucose uptake, hepatic glucose production, glycogenolysis, gluconeogenesis, lipolysis, ketogenesis, lactate turnover, amino acid metabolism, and mitochondrial substrate oxidation. Arrows indicate the direction of metabolic fluxes or regulatory influences rather than fixed quantitative relationships. The diagram is intended as a conceptual framework to illustrate how exercise modality, insulin on board, nutritional state, and counter-regulatory responses may generate different glucose trajectories despite similar continuous glucose monitoring values. Conceptual figure created by the authors with the assistance of Illustrae.

Exercise and meals do not only alter interstitial glucose; they also modify hepatic glucose production, skeletal muscle glucose uptake, glycogenolysis, gluconeogenesis, lipolysis, ketogenesis, lactate turnover, amino acid metabolism, and mitochondrial substrate oxidation [5,32]. In contrast, current AID algorithms primarily observe CGM trajectories, insulin delivery history, and user-entered information; they do not directly measure the metabolic pathways that generate those glucose profiles [7]. Consequently, the same CGM value may represent different physiological states depending on whether the user is fasting, postprandial, hyperinsulinemic, recovering from exercise, ketotic, or experiencing counter-regulatory activation [5,7,32]. Figure 1 summarizes the main metabolic pathways that may shape exercise-related glucose responses in people with type 1 diabetes using automated insulin delivery systems.

During moderate aerobic exercise, the central disturbance is often a mismatch between muscle glucose disposal and hepatic glucose availability. Contracting skeletal muscle increases glucose uptake through insulin-dependent and contraction-mediated mechanisms, including glucose transporter type 4 (GLUT4) translocation to the sarcolemma and transverse tubules [33]. At the same time, glucose delivery, capillary recruitment, intracellular phosphorylation, glycogen use, and oxidative disposal collectively determine whether increased uptake is translated into stable glycemia or hypoglycemia [34]. In individuals without diabetes, endogenous insulin secretion falls and glucagon rises, allowing hepatic glucose output to increase in parallel with muscular glucose disposal; in T1D, however, subcutaneous insulin cannot be rapidly withdrawn, and residual circulating insulin may suppress hepatic glucose production while muscle uptake remains high [32,34].

### 4.2. Hepatic Glucose Production, Counter-Regulation, and Lactate Turnover

The hepatic component is therefore central to exercise safety. Hepatic glucose production reflects the balance between glycogenolysis and gluconeogenesis, and it is modulated by insulin, glucagon, catecholamines, substrate delivery, and exercise intensity [32,34]. When insulin on board is high, hepatic glucose output may be insufficient for the increase in peripheral disposal, favoring hypoglycemia during or after aerobic activity [5,32]. Conversely, during high-intensity or interval exercise, catecholamine-mediated glycogenolysis and gluconeogenesis may exceed peripheral uptake, producing transient hyperglycemia despite ongoing insulin delivery [5,19,25,32]. This explains why carbohydrate advice cannot be based only on the current CGM value; it should also consider whether the dominant pathway is insulin-driven glucose uptake, hepatic glucose release, or post-exercise glycogen restoration [6,7,32].

Lactate is another key pathway marker rather than a nonspecific by-product of anaerobic metabolism. Contemporary lactate-shuttle theory describes lactate as an oxidative substrate, a gluconeogenic precursor, and a signaling molecule [35]. In AID users, this is clinically relevant because lactate may rise during high-intensity or mixed exercise, contribute carbon for hepatic gluconeogenesis, and coexist with transient hyperglycemia [32,35]. Therefore, a post-exercise glucose rise after intervals or resistance exercise should not automatically be interpreted as insufficient insulin; it may reflect catecholamine activation, lactate turnover, and hepatic substrate cycling [25,32,35]. This distinction is important because aggressive correction boluses after such sessions may increase delayed hypoglycemia risk if counter-regulatory drive resolves while insulin action persists [5,7,32]. From a practical perspective, post-exercise hyperglycemia after high-intensity intervals or resistance exercise should therefore be managed conservatively and individualized according to CGM trend, ketone status when relevant, insulin on board, and previous responses to similar sessions, rather than automatically treated as a simple insulin deficit [5,7,32].

### 4.3. Lipolysis, Ketogenesis, Amino Acid Turnover, and Mitochondrial Oxidation

In parallel with glucose and lactate flux, lipid-derived substrates become increasingly important as exercise duration increases or carbohydrate availability decreases. Non-esterified fatty acids and glycerol reflect adipose tissue lipolysis, whereas acylcarnitines provide information about mitochondrial fatty acid transport, beta-oxidation, and possible incomplete oxidation [36,37]. These markers are relevant for AID research because two users with similar CGM profiles may differ substantially in fuel selection: one may be relying predominantly on carbohydrate oxidation under high insulin exposure, whereas another may show greater lipid mobilization during lower insulin availability [34,36,37]. Such differences could help explain why some individuals experience delayed nocturnal hypoglycemia after prolonged aerobic sessions, while others develop prolonged post-exercise hyperglycemia after high-intensity or mixed activity [36,37,38,39].

Ketone metabolism deserves specific attention because glucose values alone do not always indicate insulin sufficiency. Insulin attenuation before exercise, prolonged fasting, low carbohydrate availability, illness, infusion-set failure, and adjunctive sodium-glucose cotransporter-2 (SGLT2) inhibitor therapy can alter the relationship between glycemia and ketogenesis [7,16,40]. In this context, hyperglycemia after pump interruption differs metabolically from postprandial hyperglycemia after an accurately delivered bolus, while euglycemia does not exclude relative insulin deficiency when carbohydrate intake is low or insulin delivery has been interrupted [7,16,40]. Therefore, beta-hydroxybutyrate or acetoacetate assessment should be considered in high-risk scenarios, particularly with unexplained hyperglycemia, nausea, illness, suspected infusion failure, prolonged insulin suspension, or low-carbohydrate intake [7,16,32,40]. From a practical perspective, ketone monitoring is particularly relevant when hyperglycemia persists despite correction, when symptoms are disproportionate to the glucose value, or when exercise is planned after prolonged insulin attenuation, fasting, illness, or suspected infusion-set failure. In these situations, exercise should generally be postponed until insulin delivery, hydration, carbohydrate availability, and ketone status have been reassessed, because euglycemia does not necessarily exclude relative insulin deficiency [7,16,40].

Amino acid turnover and tricarboxylic acid (TCA) cycle intermediates may also add mechanistic information. Alanine and glutamine can contribute to gluconeogenic substrate availability, branched-chain amino acids may reflect exercise-related proteolysis or altered oxidation, and intermediates such as citrate, malate, fumarate, and succinate provide a window into central carbon metabolism [36,37]. Although these metabolites are not currently used for routine AID decisions, their measurement in clinical studies could help distinguish whether a glucose excursion is primarily driven by carbohydrate intake, hepatic glucose release, lipid oxidation, recovery-related glycogen synthesis, or broader mitochondrial substrate handling [36,37,38,39].

### 4.4. Metabolomics-Informed Personalization of AID and Nutrition

Metabolomic studies provide the clearest bridge between AID technology and the scope of metabolic biochemistry. Untargeted metabolomics has shown that exercise in people with T1D can produce distinct metabolite responses compared with controls, including changes in lactate, TCA cycle intermediates, amino acids, glycerol, and lipid-related metabolites [36]. In addition, metabolomic, hormonal, and physiological profiling during hypoglycemic versus euglycemic exercise indicates that hypoglycemia represents a distinct stress condition rather than a simple shift in glucose concentration [35]. Fuel metabolism studies under euglycemic and hyperglycemic conditions further show that ambient glucose and insulin concentrations modify whole-body and local muscle substrate use during exercise [39], while recent targeted metabolomics suggests that acylcarnitines and the malic acid/pyruvate ratio may help distinguish exercise-related phenotypes and maximal aerobic capacity in T1D [37].

This metabolic information is especially valuable because CGM may conceal different substrate states behind similar glucose curves. For example, a falling glucose trend during steady aerobic exercise may reflect high insulin on board, suppressed hepatic glucose production, and accelerated skeletal muscle uptake [32,33,34], whereas a similar decline during recovery may be driven by glycogen resynthesis and persistent post-exercise insulin sensitivity [6,28,32]. Conversely, stable or rising glucose during high-intensity exercise may reflect catecholamine-driven hepatic output and lactate turnover rather than sufficient carbohydrate availability [19,25,32,35]. These distinctions matter because the appropriate response may differ: carbohydrate supplementation, meal-bolus reduction, exercise-target activation, avoidance of immediate correction boluses, ketone assessment, or extended monitoring may be appropriate in different metabolic contexts [7,25,32,33,34,38].

For future AID development, metabolite-informed models could support personalization in three complementary ways. First, they may identify biomarkers associated with recurrent exercise-related hypoglycemia or hyperglycemia despite apparently appropriate settings [36,37,38,39]. Second, they may help define metabolic phenotypes related to substrate availability, mitochondrial oxidation, and recovery physiology [36,37]. Third, they may guide individualized nutrition strategies by clarifying whether a person tends to rely more on carbohydrate oxidation, lipid oxidation, or counter-regulatory hyperglycemia during specific exercise modalities [34,37]. These applications remain preliminary, but they align the field with precision metabolism and reinforce the need to integrate CGM, insulin delivery logs, diet records, activity metrics, and metabolomic sampling in future trials [36,37,38,39]. Because similar glucose patterns may arise from different underlying metabolic states, Table 2 summarizes the main metabolic pathways, representative biomarkers, and their mechanistic relevance for interpreting nutrition and exercise challenges in AID users.

## 5. Nutritional Strategies Around Physical Activity

Within this metabolic framework, nutritional management remains essential for people with T1D using AID systems. Although AID can automate aspects of basal insulin delivery and correction behavior, it cannot fully compensate for inaccurate carbohydrate estimation, poorly timed boluses, or unannounced meals. Carbohydrate intake before, during, and after exercise should therefore be interpreted in relation to current glucose, CGM trend, insulin on board, timing of the last meal, exercise intensity, and the behavior of the specific algorithm. Commercial systems differ in how rapidly they attenuate insulin and whether they provide activity modes, temporary targets, or more aggressive automated corrections [6,7].

The first practical implication is that accurate carbohydrate counting remains a major determinant of postprandial glycemia. In children and adolescents using pump therapy, errors in carbohydrate estimation were associated with postprandial glucose deviations [41]. This evidence remains relevant in the AID era because most HCL systems still require meal announcements and user-entered carbohydrate values. Large underestimation can produce prolonged hyperglycemia because subcutaneous insulin action is delayed, whereas overestimation may precipitate hypoglycemia, especially when activity occurs soon after the meal [6,7,41].

When exercise is planned, pre-exercise carbohydrate intake is most useful when glucose is low-normal or falling, circulating insulin levels are high, or planned activity is aerobic and prolonged. However, carbohydrate supplementation should not be applied mechanically. If the system has already reduced insulin delivery or if the user begins exercise with low insulin on board, excessive carbohydrate may cause hyperglycemia. Conversely, if activity begins soon after a meal bolus, even moderate exercise may require both bolus reduction and carbohydrate support. The evidence favoring announced postprandial exercise with meal bolus reduction is particularly relevant for this scenario [6,7,25].

This is also why the timing of activity-mode activation is clinically relevant. If an exercise target is selected only when glucose is already falling, the system may reduce future insulin delivery but cannot reverse circulating insulin that was delivered earlier. By contrast, selecting the target sufficiently in advance may reduce insulin on board before exercise begins, although the optimal timing varies according to the system, the planned activity, the recent bolus history, and the individual response. Thus, nutritional recommendations should be coordinated with algorithmic settings rather than treated as independent decisions [6,7,25].

During the exercise bout, carbohydrate intake may still be required despite the use of activity mode or temporary targets, especially during prolonged aerobic activity. However, the amount needed varies substantially between individuals, supporting individualized rather than fixed prescriptions. In contrast, routine carbohydrate intake before short, high-intensity, or resistance-based sessions may be unnecessary and may worsen exercise-associated hyperglycemia. After exercise, nutritional planning should consider delayed insulin sensitivity, glycogen repletion, training load, and sleep timing; the biphasic increase in glucose requirements after afternoon exercise illustrates that recovery risk may extend for several hours [6,7,28].

A useful way to interpret carbohydrate intake during exercise is to distinguish between prevention, stabilization, and rescue. Preventive carbohydrate is used before a predictable glucose decline, stabilizing carbohydrate is given in small amounts during prolonged activity to match ongoing substrate use, and rescue carbohydrate is used when hypoglycemia is already present or imminent. This distinction is important because excessive preventive intake may trigger automated insulin delivery or correction behavior, whereas delayed rescue intake may expose the user to symptomatic hypoglycemia and subsequent rebound hyperglycemia. Therefore, CGM trend arrows, symptoms, insulin on board, and the expected intensity of the session should be interpreted together [6,7,28].

Meal timing adds another layer of complexity. Exercise performed in the postabsorptive state may require less meal-bolus manipulation, although prolonged activity can still lead to hypoglycemia. By contrast, exercise performed shortly after a meal requires more anticipatory management because the algorithm cannot remove previously delivered prandial insulin. Practical options include reducing the meal bolus, activating an exercise target in advance, delaying intense activity until insulin action is lower, or using smaller carbohydrate doses guided by CGM trend arrows and symptoms [6,7,28,41].

In addition to carbohydrate timing, macronutrient composition adds further complexity. High-fat meals may delay gastric emptying and prolong postprandial hyperglycemia, while high-protein meals can contribute to delayed glucose rises, particularly when carbohydrate intake is modest [8]. In children and adolescents with T1D, dietary protein and fat increased late postprandial glucose excursions from approximately 3 to 5 h after the meal, and their effects were additive [8]. Although this landmark study was not conducted in AID users, it remains physiologically relevant because current HCL systems still rely on subcutaneous insulin delivery and user-entered meal information. Evidence directly addressing high-fat or high-protein mixed meals followed by exercise in AID users remains sparse. Therefore, in current practice, mixed-meal management in AID users should rely on individualized CGM pattern review, careful interpretation of delayed postprandial excursions, and platform-specific bolus or algorithm features rather than universal macronutrient formulas. Diet quality and structured lifestyle education remain relevant in T1D management, even when technology improves glycemic metrics [42,43].

From a dietary-pattern perspective, the quality and structure of meals may also affect the sustainability of AID use. A system that improves glycemic metrics could unintentionally encourage dietary flexibility without adequate education, but the available evidence does not support assuming that advanced HCL use necessarily worsens energy or nutrient intake [44]. Nevertheless, individuals who repeatedly rely on automated corrections after imprecise carbohydrate counting may experience greater glycemic variability, higher insulin exposure, and more device alarms. For this reason, AID education should continue to include carbohydrate estimation, meal timing, recognition of high-fat and high-protein meal patterns, and interpretation of postprandial CGM curves [8,41,42,43,44].

Because manual meal entry remains burdensome, automated meal detection has been explored as a strategy to reduce dependence on meal announcements. Machine learning approaches can detect meal-related glucose rises from CGM data [45], and in silico studies suggest that alternative insulin delivery routes, such as intraperitoneal insulin, could reduce the need for meal announcement by better approximating physiological insulin kinetics [46]. However, these approaches must distinguish meals from exercise recovery, stress hyperglycemia, sensor noise, illness, and other non-meal excursions. Fully automated dietary management therefore remains a future goal rather than a current clinical standard [45,46].

**Figure 2:**
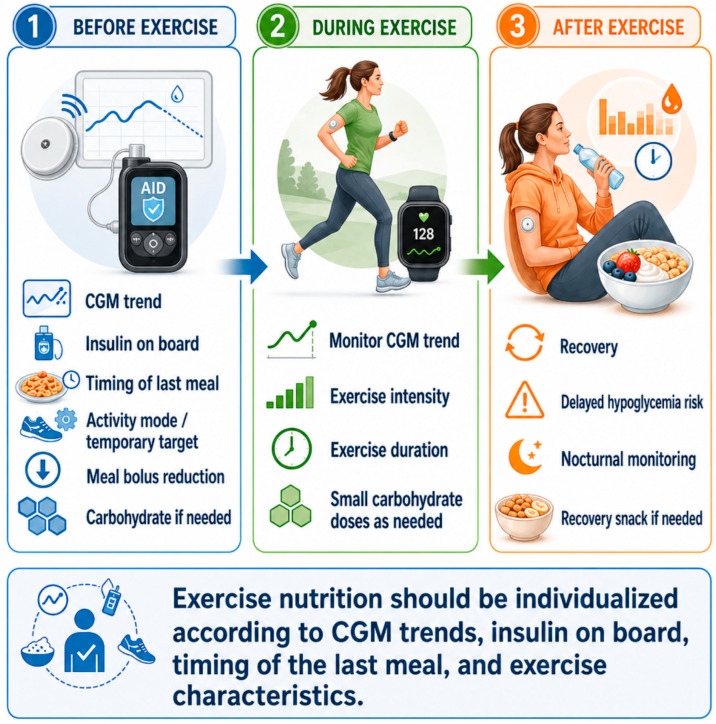
Nutritional strategies before, during, and after exercise in people with type 1 diabetes using automated insulin delivery systems. The figure summarizes practical decision points related to pre-exercise preparation, carbohydrate intake, exercise-target or temporary-target activation, continuous glucose monitoring trend interpretation, insulin on board, intra-exercise monitoring, and post-exercise recovery. The arrows represent the temporal sequence of decision-making rather than a universal protocol, as the optimal strategy depends on the AID platform, exercise modality, recent bolus insulin, meal timing, and individual glucose patterns. Conceptual figure created by the authors with the assistance of Illustrae. CGM, continuous glucose monitoring.

Overall, nutritional strategies in AID users should be individualized through repeated pattern recognition rather than converted into fixed carbohydrate rules. Reviewing CGM profiles together with bolus timing, activity-mode use, exercise type, symptoms, and recovery glucose patterns can help identify whether a person tends to require earlier insulin reduction, smaller carbohydrate doses during activity, a bedtime recovery snack, or more conservative correction behavior after intense sessions. This approach links nutrition education to the actual behavior of the algorithm and to the user’s own metabolic responses [7,25,28,41]. Based on these considerations, Table 3 summarizes the main nutritional strategies that may be used around exercise in AID users, indicating when each approach may be useful, its main limitations, and its clinical interpretation. Figure 2 summarizes the main nutritional considerations around exercise, including pre-exercise preparation, glucose monitoring during activity, and post-exercise recovery management.

## 6. Meal Announcement, Macronutrient Complexity, and Algorithmic Personalization

This nutritional complexity explains why the continuing need for meal announcement remains one of the clearest examples of the interaction between user behavior and algorithmic control. AID systems can reduce basal insulin errors and respond to glucose trends, but most still require users to estimate carbohydrate intake and deliver meal boluses. This is especially difficult when meals are followed by exercise, because the system must manage both the incoming glucose rise and the subsequent increase in muscle glucose uptake [25,41].

In response, proof-of-concept decision-support approaches based on adaptive algorithms and reinforcement learning have been explored to personalize meal-related insulin dosing. Reinforcement learning has been tested for individualized insulin dosing in high-fat meals followed by aerobic exercise, suggesting that computational models may learn personal response patterns beyond static rules [48]. Similarly, deep reinforcement learning bolus calculators and multi-agent reinforcement learning basal-bolus advisors have been proposed as experimental strategies to reduce dependence on manual carbohydrate counting and coordinate basal-prandial decisions [49,50]. However, these approaches should currently be interpreted as emerging research tools rather than fully established components of routine AID care.

The relevance of these approaches is greater in exercise contexts than in standard postprandial control, because the optimal response may involve both insulin and carbohydrate decisions. For example, a system trained only to reduce postprandial hyperglycemia may recommend more insulin after a high-fat meal, whereas the same meal followed by planned aerobic exercise could require a more conservative bolus strategy and earlier exercise-target activation. Adaptive algorithms therefore need to learn not only the glycemic effect of meals but also the conditional interaction among meal composition, insulin action, physical activity, and recovery physiology [25,48,50].

Nevertheless, the clinical applicability of these approaches remains preliminary. Many artificial intelligence models are trained in simulated environments, small proof-of-concept trials, or highly selected datasets. Performance may deteriorate with irregular meals, missed announcements, menstrual-cycle variation, illness, stress, alcohol intake, or spontaneous exercise. Clinical translation will require transparent safety constraints, regulatory evaluation, and decision pathways that users and clinicians can understand. In practice, an algorithm is clinically valuable only if its behavior is both accurate and interpretable [48,50].

Interpretability is especially important when recommendations involve nutrition. Users may accept an automated insulin reduction more readily than an unexplained carbohydrate recommendation or a suggestion to avoid correction insulin before activity. Consequently, future systems should aim to communicate the rationale for recommendations in clinically meaningful terms, such as high insulin on board, rapidly falling glucose, recent bolus exposure, expected aerobic activity, or previous nocturnal hypoglycemia after similar sessions. Such transparency could improve trust while helping users learn the metabolic logic behind repeated algorithmic decisions [48,50,51,52,53].

Beyond algorithms, adjunctive pharmacological and delivery strategies may also influence postprandial control. Empagliflozin, SGLT2 inhibitor, has improved TIR and reduced glycemic variability when added to closed-loop insulin delivery in T1D [40]. Nevertheless, SGLT2 inhibitors increase ketone and DKA risk in T1D, so they require careful selection, education, and ketone monitoring. Alternative insulin delivery approaches, such as Technosphere inhaled insulin, have also been studied as a way to mimic first-phase insulin secretion and reduce postprandial excursions in automated systems [54].

These examples also illustrate why postprandial innovations should be evaluated in metabolically realistic contexts. A strategy that performs well after a standardized meal may behave differently when the meal is high in fat or protein, when exercise is started shortly after eating, or when insulin delivery has been reduced to prevent hypoglycemia. Therefore, future studies of adjunctive therapies and alternative insulin routes should report not only postprandial TIR but also ketone safety, hypoglycemia after activity, correction requirements, and user burden [40,54].

Accordingly, advanced algorithms and adjunctive strategies should be interpreted as supports for decision-making rather than substitutes for nutritional literacy. They may reduce the burden of carbohydrate counting and improve postprandial control, but users still need to understand when a system may fail, how exercise modifies expected insulin responses, and when persistent hyperglycemia or hypoglycemia requires manual action.

## 7. Technological Adaptations and Emerging Directions

The same principle applies to future AID development: progress in exercise and nutrition management will depend on better anticipation of metabolic disturbances, not only on stronger correction of established glucose excursions. Current systems perform well when glucose changes gradually, but they are less reliable when meals, exercise, illness, stress, and recovery overlap. Several adaptations are being investigated, including multivariable control, additional physiological sensors, dual-hormone delivery, adaptive algorithms, simulation platforms, digital twins, and glucose-responsive insulin technologies.

One strategy is to expand the number of physiological inputs available to the algorithm. Multivariable control strategies can incorporate physiological and behavioral inputs to modify insulin delivery around physical activity [55]. Wearable sensors are particularly relevant because heart rate, accelerometry, and activity trackers may detect exercise onset and intensity before CGM reflects interstitial glucose decline [26,27]. The key challenge is not only detecting movement but interpreting its glycemic meaning: resistance exercise, steady aerobic exercise, interval training, and everyday activity may generate different glucose trajectories despite similar sensor signals [55].

In parallel, dual-hormone systems remain attractive because glucagon can counterbalance hypoglycemia without requiring carbohydrate ingestion. Positive findings from exercise and overnight studies support their potential [19,20,21], and trial protocols continue to evaluate dual-hormone fully closed-loop systems under real-world conditions [56]. Widespread adoption will require stable glucagon formulations, reliable infusion hardware, acceptable cost, and algorithms that prevent both under-treatment of hypoglycemia and excessive glucagon delivery [56].

Before such systems were tested widely, simulation and virtual populations remained important for safer algorithm development. Exercise-integrated virtual patient populations can model diverse responses to physical activity and test robustness across scenarios that are difficult to reproduce clinically [57]. The University of Virginia/Padova simulator has become a central platform for in silico testing of glucose control algorithms and continues to be refined [58]. Digital twin approaches represent a further emerging step, as they aim to generate patient-specific models that could support individualized decision-making and improve glycemic outcomes [59]. However, their use in AID-related nutrition and exercise management should still be considered exploratory, particularly because external validation in diverse free-living populations remains limited. In addition, generative models and glycemia prediction algorithms may improve the creation of realistic glucose time series and short-term forecasting [60,61].

However, models used for AID development should incorporate exercise and nutrition with sufficient physiological detail. A virtual patient that reproduces fasting glucose-insulin dynamics may still fail to capture postprandial exercise, delayed gastric emptying after high-fat meals, menstrual-cycle-related insulin changes, or prolonged recovery after glycogen-depleting sessions. Therefore, simulation platforms and digital twins should be validated not only against average CGM metrics but also against context-specific outcomes such as hypoglycemia during exercise, nocturnal risk after activity, postprandial excursions after mixed meals, and the number of user interventions required [60,61].

At the longer-term frontier, other technologies could alter the field more fundamentally. Smart insulin and glucose-responsive analogues aim to adjust insulin activity according to ambient glucose, potentially reducing reliance on external control loops [62]. Broader innovation frameworks also compare AID devices with biological approaches such as cell therapy, suggesting that future care may combine technological and biological strategies to reduce user burden and restore more physiological glucose regulation [63].

Accordingly, future systems should not be judged only by mean TIR. In exercise and nutrition contexts, decisive outcomes include time below range during and after activity, postprandial excursions after mixed meals, ketone safety, metabolic recovery, number of user interventions, alarm burden, confidence during exercise, and the ability to manage unannounced events safely. A system that improves average glycemic metrics but requires frequent corrections, repeated alerts, or complex user decisions may still be difficult to sustain in daily life. To provide an overview of current and emerging technological solutions, Table 4 summarizes the main adaptations that may improve nutrition and exercise management in AID systems, together with their potential contribution, current limitations, and supporting evidence.

## 8. Special Populations and Interindividual Variability

This need for personalization is especially evident in populations with marked metabolic variability. AID performance is influenced by age, developmental stage, sex, pregnancy, comorbidities, body composition, and behavioral context, because all these factors can modify insulin sensitivity, substrate availability, counter-regulatory responses, and the relationship between insulin delivery and glucose appearance. Children and adolescents are a central example: eating patterns and physical activity are often unpredictable, insulin sensitivity may be higher, and caregiver involvement is frequently required. Closed-loop therapy has shown clinically relevant benefits in very young children, including improved glycemic control over 16 weeks compared with sensor-augmented pump therapy [64]. Pediatric reviews also emphasize that children require developmentally adapted algorithms, education, and family support rather than direct extrapolation from adult data [65]. In newly diagnosed youth, early closed-loop initiation improved glycemic control but did not prevent the decline in residual C-peptide secretion, indicating that metabolic benefits should be distinguished from disease-modifying effects [66].

Pregnancy and hormonal variation represent additional metabolically demanding contexts. Pregnancy involves rapidly changing insulin resistance, tighter glycemic targets, altered meal patterns, nausea, and fetal safety considerations, all of which can affect the performance of AID systems [67,68]. More recent evidence from the AiDAPT trial showed that hybrid closed-loop therapy can improve maternal glycemic control during pregnancy complicated by T1D. Nevertheless, pregnancy-specific AID use should still be interpreted as a highly dynamic metabolic context, because insulin requirements change markedly across gestation and postpartum, and nutritional decisions are constrained by nausea, meal tolerance, fetal safety, and stricter glycemic targets [69].

Outside pregnancy, menstrual-cycle-related changes in insulin requirements may also influence glycemic control, particularly when exercise and nutrition vary across the cycle [70]. These examples reinforce that sex-specific and life-stage factors should not be treated as secondary variables, because they may directly alter insulin needs, glucose flux, and the risk of hypo- or hyperglycemia.

Highly trained athletes and endurance sport participants represent another subgroup in whom standard AID recommendations may be insufficient. Prolonged aerobic exercise, repeated training sessions, competition stress, variable carbohydrate availability, dehydration, travel, and altered sleep may produce glucose patterns that differ from those observed in short laboratory protocols. In these users, management should prioritize individualized planning around insulin on board, timing of exercise-target activation, carbohydrate periodization, post-exercise recovery, and delayed nocturnal hypoglycemia risk [32]. Alcohol intake should also be considered in active AID users, particularly after evening exercise, because alcohol can impair hepatic gluconeogenesis and counter-regulatory responses, thereby increasing the risk of delayed hypoglycemia [71].

Comorbidities and body composition further modify the metabolic context in which AID systems operate. In advanced chronic kidney disease, altered insulin clearance and increased hypoglycemia risk may affect algorithm behavior, whereas obesity in T1D may increase insulin requirements, insulin resistance, cardiometabolic risk, and the complexity of weight management [72,73,74]. Adjunctive treatments such as tirzepatide have shown preliminary potential for reducing weight and insulin requirements in adults with T1D, but evidence remains early and careful safety evaluation is needed [75]. Therefore, AID recommendations should not be applied uniformly across populations with different metabolic profiles.

Highly active individuals highly trained athletes, and endurance sport participants may require particularly individualized AID strategies because training load, competition timing, prolonged aerobic exercise, repeated training sessions, recovery nutrition, glycogen restoration, variable carbohydrate availability, dehydration, travel, altered sleep, and performance goals may conflict with standard algorithmic assumptions. In these users, management should prioritize individualized planning around insulin on board, timing of exercise-target activation, carbohydrate periodization, sensor interpretation, ketone assessment when relevant, post-exercise recovery, and delayed nocturnal hypoglycemia risk. Thus, the most useful clinical approach is iterative: reviewing CGM, insulin delivery, nutrition, exercise type, and recovery patterns after repeated sessions to identify individual metabolic responses [7,51,71].

Importantly, interindividual variability is not only biological. Two users with similar insulin sensitivity and similar exercise routines may experience AID differently depending on confidence, health literacy, access to clinical advice, family support, and previous experiences of severe hypoglycemia. Around exercise, fear and uncertainty may lead either to activity avoidance or to excessive carbohydrate correction. A clinically effective AID strategy should therefore integrate behavioral support with metabolic and algorithmic personalization [51,52].

## 9. Psychosocial and Implementation Considerations

These population-specific considerations lead directly to psychosocial and implementation issues, because the clinical value of AID depends on how users interpret and act on metabolic information in daily life. Fear of hypoglycemia is a major barrier to physical activity in people with T1D and may persist even when technology improves objective glycemic outcomes [51,52]. In practice, this fear may lead to exercise avoidance, excessive preventive carbohydrate intake, conservative insulin dosing, or delayed correction of hyperglycemia. Therefore, psychosocial responses can directly influence substrate availability, insulin exposure, and post-exercise glycemic patterns.

Trust is also central to sustained AID use. Experienced users describe trust as dynamic, developing through repeated interactions with the system and potentially being disrupted by unexplained glucose excursions, infusion failures, sensor errors, or algorithmic decisions that conflict with user expectations [53]. Adolescents using smartphone-hosted closed-loop algorithms have also reported that technology can increase freedom while requiring new forms of attention and responsibility in everyday life [76]. These findings suggest that quality-of-life benefits should be interpreted alongside workload, expectations, alarm burden, and the user’s ability to understand when carbohydrate intake, insulin adjustment, ketone testing, or exercise-target activation is required [77].

At the implementation level, device adoption is shaped by access, education, cost, digital literacy, alarm burden, skin issues, and clinician confidence [78]. Open-source AID systems illustrate both the empowerment of technically engaged users and the challenges of support, regulation, and liability [79]. Practical implementation guidance emphasizes that device choice, training, follow-up, and troubleshooting must be individualized, particularly regarding infusion failure, ketone monitoring, hyperglycemia management, carbohydrate counting, insulin on board, and the safe use of activity modes [80,81].

Health-care teams also require support, because rapidly changing device features, data overload, limited consultation time, and uneven access to specialist training can impair clinical interpretation [82,83,84]. Future AID systems should therefore be evaluated not only by glycemic performance, but also by usability, exercise robustness, interpretability, alarm burden, and their ability to support daily metabolic decisions [85,86]. Implementation should be understood as a clinical intervention rather than a one-time device prescription: follow-up should review TIR, time below range, nocturnal patterns, meal-related excursions, exercise confidence, user-reported burden, and recurrent situations in which nutrition or insulin decisions remain difficult.

## 10. Research Gaps and Reporting Priorities

The interpretation of the available evidence is limited by substantial heterogeneity across studies. Existing trials and observational studies differ in AID platform, algorithm behavior, exercise modality, intensity and duration, timing of exercise in relation to meals, insulin on board, nutritional strategy, carbohydrate dose, population characteristics, and outcome windows. In addition, many studies are small, short-term, or conducted under controlled laboratory conditions, whereas free-living studies capture more realistic behavioral variability but often rely on less standardized exercise and dietary data. These differences limit direct comparisons across systems and prevent firm conclusions regarding a single optimal strategy for all AID users.

Another limitation is the inconsistent reporting of algorithm-level performance metrics. Studies evaluating exercise detection, meal detection, or sensor-assisted decision support often report glycemic outcomes such as TIR, TBR, or TAR, but do not consistently provide false-positive rates, false-negative rates, detection latency, missed-event frequency, or the number of user overrides required. These metrics are essential to determine whether an algorithm is clinically usable in real-world conditions, where exercise, meals, stress, illness, and sensor noise may generate overlapping signals. Future studies should therefore report both glycemic outcomes and algorithm-performance metrics, including detection accuracy, timing of detection, safety constraints, and user burden.

Taken together, the field is moving from reactive glucose correction toward anticipatory and personalized metabolic control, but this transition remains incomplete. Current evidence supports the benefits of AID for average glycemic outcomes, yet fewer studies evaluate how systems perform when meals, exercise, insulin on board, menstrual-cycle variation, stress, illness, and recovery overlap. Future trials should therefore move beyond isolated device efficacy and examine the complete human-technology-nutrition-exercise-metabolism interface [7,18,27,28,29,30,38,39,40,43,44,45,46,47,48,49,62,63,64,65,66,67,68,69,70,71,72,73,74,75].

One priority is to design trials that deliberately combine metabolic stressors rather than isolating them. Many studies test meals, exercise, or algorithm performance separately, but daily life frequently combines all three. For instance, a user may consume a mixed meal, deliver a reduced bolus, start unplanned activity, experience automated insulin attenuation, and then enter a period of nocturnal recovery. Studies that reproduce these overlapping scenarios would be more informative for clinical practice than protocols restricted to standardized meals or isolated exercise bouts [6,7,18,21,36,37,38,39,40,43,44,45,46,47,48,49].

Accordingly, several reporting priorities emerge from this synthesis. Exercise studies should report modality, intensity, duration, timing in relation to meals, insulin on board, use and timing of activity mode, carbohydrate intake before and during activity, recovery nutrition, and nocturnal outcomes. Nutrition studies should characterize carbohydrate estimation accuracy, macronutrient composition, meal timing, bolus timing, automated corrections, and postprandial exercise. In addition, metabolic studies should incorporate, where feasible, lactate, ketone bodies, non-esterified fatty acids, acylcarnitines, insulin levels, counter-regulatory hormones, and recovery biomarkers [6,7,18,27,28,29,30,31,36,37,38,39,40].

Metabolomics studies also need more consistent reporting of sampling timing. A single post-exercise blood sample may miss early lactate responses, delayed lipolysis, ketone changes, amino acid turnover, or recovery-related shifts in acylcarnitines and tricarboxylic acid cycle intermediates. Future work should therefore align metabolite sampling with CGM, insulin delivery logs, meal timing, exercise intensity, and recovery outcomes. This would make it possible to determine whether specific metabolite patterns predict recurrent hypoglycemia, prolonged hyperglycemia, or different carbohydrate requirements in AID users [27,28,29,30].

Moreover, standardization is needed in outcome selection. TIR is essential but insufficient when studying exercise and nutrition. Time below range during and after activity, time above range after mixed meals, glycemic variability, DKA risk, ketone status, number of manual interventions, alarm burden, treatment satisfaction, sleep, exercise confidence, and quality of life should be reported more consistently. This broader outcome framework would make AID research more relevant to daily decision-making and to metabolically complex free-living contexts [7,27,28,29,30,62,63,64,65,66,67,68,69,70,71,72,73,74,75].

A second priority is to evaluate user burden as an outcome rather than assuming that improved automation automatically reduces workload. An AID strategy may improve TIR but still require repeated meal edits, manual corrections, exercise announcements, alarm responses, or preventive carbohydrate intake. Conversely, a slightly less aggressive algorithm may be preferable for some users if it reduces hypoglycemia fear and supports regular physical activity. Future studies should therefore combine CGM-derived outcomes with behavioral and psychosocial measures that capture how people actually use these systems [62,63,64,65,66,67,69,70,71,72,73,74,75].

Finally, equity and implementation should be integrated into research design. AID benefits will be limited if access depends on socioeconomic status, technical literacy, specialist availability, or health-system reimbursement. Studies should therefore include diverse populations, evaluate scalable education models, and report the training and clinical support needed to achieve successful use. Personalized AID will only be clinically meaningful if it can be implemented safely and equitably [67,68,69,70,71,72,73]. In line with these limitations, Table 5 summarizes the main research and implementation priorities needed to improve the integration of AID technology, nutrition, exercise, and metabolic monitoring in future studies and clinical practice.

## 11. Conclusions

In conclusion, AID systems have substantially improved T1D management, but exercise and nutrition remain complex metabolic contexts in which automation is not yet fully autonomous. Available evidence supports individualized carbohydrate strategies, meal bolus adjustment before postprandial exercise, exercise announcement when possible, appropriate use of activity modes or temporary targets, and careful post-exercise monitoring, particularly when activity is aerobic, prolonged, or performed with high insulin on board. These strategies should be interpreted through a broader metabolic framework that considers substrate availability, hepatic glucose production, lactate turnover, ketone risk, lipid mobilization, glycogen repletion, mitochondrial fuel handling, and behavioral feasibility. Future advances may arise from activity sensors, adaptive algorithms, dual-hormone delivery, digital twins, automated meal detection, glucose-responsive insulin technologies, and metabolomics-informed personalization; however, several of these approaches remain exploratory or supported mainly by proof-of-concept, simulation, or early clinical evidence. Therefore, they require validation in free-living settings and should be evaluated not only for TIR but also for safety, usability, interpretability, equity, user burden, and support for real-world metabolic decision-making. Overall, AID should be presented as a powerful support for metabolic self-management rather than as a replacement for nutrition education, exercise planning, and individualized clinical follow-up.

## Figures and Tables

**Table 1:** Core exercise-related challenges for AID systems and practical implications.

Challenge	Dominant Mechanism	Clinical Implication for AID Users	Representative Evidence and Evidence Type
Moderate aerobic exercise	Increased skeletal muscle glucose uptake in the presence of circulating insulin	Consider advance activity mode or temporary target when available; adjust carbohydrate according to CGM trend and insulin on board.	Refs. [5,6,7,25,28]; physiological, consensus, and AID exercise evidence
High-intensity or interval exercise	Counter-regulatory activation, lactate production, and variable hepatic glucose release	Avoid a universal carbohydrate rule; monitor for early hyperglycemia and later glucose decline.	Refs. [5,24,25]; physiological and clinical exercise evidence
Postprandial exercise	Overlap between prandial insulin action and exercise-induced glucose uptake	Planned exercise announcement and meal bolus reduction may be more effective than relying only on closed-loop automation.	Refs. [6,7,25]; consensus and randomized/crossover AID evidence
Unannounced spontaneous activity	Algorithm responds after glucose begins to change or after activity is detected	Remains a key limitation of current HCL systems; sensor-based activity detection may reduce but not eliminate risk.	Refs. [24,26,27]; free-living observational and sensor-detection evidence
Delayed nocturnal hypoglycemia	Exercise-related increase in insulin sensitivity and glycogen restoration during recovery	Post-exercise monitoring, bedtime strategies, target adjustment, or low-dose glucagon may be needed in selected users.	Refs. [17,28,29]; clinical trial, physiological, and consensus evidence

AID, automated insulin delivery; CGM, continuous glucose monitoring; HCL, hybrid closed loop.

**Table 2:** Metabolic pathways, representative biomarkers, and mechanistic interpretation of nutrition and exercise challenges in AID users.

Metabolic Domain	Representative Biomarkers and Associated Physiological Pathways	Relevance for AID Users	Research or Clinical Implication
Glycolytic flux	Lactate, pyruvate, glucose uptake; glycolysis, lactate shuttle, skeletal muscle glucose disposal, and lactate-supported hepatic gluconeogenesis	May explain transient glucose instability during high-intensity or mixed exercise.	Report exercise intensity and consider lactate-related patterns when interpreting post-exercise hyperglycemia [36,37,38,39].
Hepatic glucose production	Glycogenolysis, gluconeogenesis, glucagon, catecholamines, cortisol, and growth hormone; hepatic glucose output and counter-regulatory activation	Determines whether exercise leads to glucose decline or counter-regulatory hyperglycemia.	Interpret exercise announcement and bolus reduction according to insulin on board and expected hepatic response [5,25].
Lipid mobilization and oxidation	Non-esterified fatty acids, glycerol, acylcarnitines; adipose tissue lipolysis, mitochondrial fatty acid transport, beta-oxidation, and incomplete fatty acid oxidation	May influence recovery metabolism and delayed glucose responses after prolonged activity.	Future trials should include lipid-related biomarkers and recovery outcomes when feasible [36,37].
Ketone metabolism	Beta-hydroxybutyrate, acetoacetate; ketogenesis, hepatic fatty acid oxidation, and relative or absolute insulin deficiency	Relevant when insulin delivery is reduced or interrupted, or when carbohydrate intake is restricted.	Ketone monitoring should be considered in high-risk situations, including illness, pump failure, or adjunctive therapy [7,16,40].
Central carbon metabolism	Citrate, malate, fumarate, succinate, malic acid/pyruvate ratio; tricarboxylic acid cycle activity, anaplerosis, and mitochondrial substrate oxidation	May distinguish metabolic phenotypes and aerobic capacity in T1D.	Metabolomic profiling may support future digital twins and personalized nutrition-exercise algorithms [36,37].

AID, automated insulin delivery; T1D, type 1 diabetes.

**Table 3:** Nutritional strategies for exercise in AID users.

Strategy	When It May Help	Representative Dose or Approach Reported in Studies	Main Caution	Clinical Interpretation
Pre-exercise carbohydrate	Low-normal or falling glucose, aerobic exercise, prolonged activity, or high insulin on board	Scheduled snacks of 15 g carbohydrate every 30 min during prolonged hiking; spontaneous pre-exercise carbohydrate intake in free-living studies was commonly around 20 g when reported [30]	Excess carbohydrate may cause hyperglycemia if insulin delivery has already been reduced.	Dose should be guided by CGM trend, recent bolus timing, and exercise type [6,7]
Meal bolus reduction before activity	Planned exercise within a few hours after a meal	Postprandial exercise protocols have used standardized meals of approximately 65 g carbohydrate with meal bolus reductions of around 33%; other protocols used a 25% bolus reduction with pre-exercise carbohydrate [47]	Excessive reduction may cause postprandial hyperglycemia.	Often most effective when combined with exercise announcement or activity mode [25]
Carbohydrate during exercise	Longer aerobic sessions or persistent downward glucose trends	Fractionated low-glycemic-index carbohydrate dosing of 0.25 g/kg during exercise has been evaluated; automated systems have also suggested 15 g rapid-acting carbohydrate when hypoglycemia was predicted [38]	Fixed carbohydrate rules may not match individual insulin sensitivity.	Small repeated doses may be preferable to large reactive intakes [28]
Post-exercise recovery snack or target adjustment	Afternoon/evening exercise, delayed hypoglycemia risk, or high training load	Rescue carbohydrate in free-living AID studies has been reported around a median of 25.5 g when hypoglycemia occurred [12]	May be unnecessary after short or predominantly anaerobic sessions.	Should be guided by prior nocturnal CGM patterns and training load [28,29]
Macronutrient-aware meal planning	High-fat or high-protein meals, late meals, or exercise after mixed meals	No universal AID-specific dose is established; delayed postprandial effects of fat and protein have been described mainly outside AID-specific exercise studies [8]	Evidence specifically in AID-exercise settings remains limited.	CGM pattern review is currently more practical than universal formulas [42,43,44]
Automated meal detection support	Missed or inaccurate meal announcements	Diabeloop DBLG1 has calculated preventive carbohydrate recommendations averaging 41.1 g on days with physical activity; SAFE-AP suggested 15 g rapid-acting carbohydrate when hypoglycemia was predicted [14]	Meals must be distinguished from stress, exercise recovery, and sensor noise.	Promising but not yet a replacement for user-entered meal information [45,46]

AID, automated insulin delivery; CGM, continuous glucose monitoring.

**Table 4:** Technological adaptations relevant to nutrition and exercise management.

Approach	Potential Contribution	Current Limitation	Relevant Evidence
Activity sensors	Earlier detection of exercise onset and intensity	False positives and uncertain interpretation of mixed activities	[26,27]
Multivariable control	Integration of physiological and behavioral inputs around activity	Requires robust validation across spontaneous and planned exercise scenarios	[55]
Dual-hormone AID	Glucagon delivery may reduce exercise-related hypoglycemia	Device complexity, glucagon dosing, cost, rebound hyperglycemia, and long-term usability	[56]
Automated meal detection	Reduced burden of meal announcements	Difficulty distinguishing meals from non-meal glucose excursions	[45,46]
Adaptive or artificial intelligence-based algorithms	Emerging decision-support for complex meals and exercise	Mostly proof-of-concept evidence; limited real-world validation, safety constraints, and explainability	[48,49,59,60,61]
Simulation and digital twins	Safer preclinical testing and exploratory individualized scenario evaluation	Need for external validation across diverse free-living populations	[57,58,59]
Glucose-responsive insulin technologies	Potential long-term reduction in dependence on external control loops	Still early-stage compared with commercial AID systems	[62]

AID, automated insulin delivery.

**Table 5:** Research and implementation priorities for AID, nutrition, exercise, and metabolism.

Priority	Rationale	Suggested Direction
Personalized exercise guidance	Responses vary by exercise modality, insulin on board, meal timing, and fitness.	Develop decision aids that combine CGM trend, insulin on board, meal timing, and activity type [7,86].
Meal-exercise interaction studies	Postprandial exercise remains a high-risk scenario for hypoglycemia.	Test bolus reduction, activity mode timing, and carbohydrate strategies under standardized protocols [25]
Macronutrient-focused trials	Fat and protein may cause delayed excursions, especially around exercise.	Study high-fat and high-protein meals in AID users with and without exercise [42,43,44,48]
Sensor-based automation	Unannounced activity remains a key limitation of current HCL systems.	Validate heart rate, accelerometry, and other wearable signals under free-living conditions [26,27,55]
Psychosocial outcomes	Trust, fear, and burden influence long-term adoption.	Include quality of life, diabetes distress, sleep, and exercise confidence in trials [51,52,53,76,77,78]
Equitable implementation	Access and training determine who benefits from AID.	Improve reimbursement, clinician education, interoperability, and culturally adapted support [78,79,80,81,82,83,84]
Metabolic biomarker reporting	Glucose metrics alone cannot distinguish hepatic, muscular, lipid, or ketone-related mechanisms.	Include lactate, ketones, lipid-related markers, insulin on board, and recovery variables when feasible [36,37,38,39]
Metabolomics-informed personalization	Interindividual variability may reflect distinct metabolic phenotypes.	Explore targeted and untargeted metabolomics to develop digital twins and personalized nutrition-exercise algorithms [36,37,59]

AID, automated insulin delivery; CGM, continuous glucose monitoring; HCL, Hybrid closed loop.

## Data Availability

No new data were created or analyzed in this study. Data sharing is not applicable.

## Abbreviations

The following abbreviations are used in this manuscript:

AID	Automated insulin delivery
CGM	Continuous glucose monitoring
DKA	Diabetic ketoacidosis
GLUT4	Glucose transporter type 4
HCL	Hybrid closed loop
SGLT2	Sodium-glucose cotransporter-2
T1D	Type 1 diabetes
TCA	Tricarboxylic acid
TIR	Time in range
